# Supplementary material for: Pregnancy loss and its predictors among ever-pregnant women in Sub-Saharan Africa: Multilevel mixed effect negative binomial regression
Source: PLOS Glob Public Health. 2025 Apr 7;5(4):e0004316. doi: 10.1371/journal.pgph.0004316 (PMC11975090; doi:10.1371/journal.pgph.0004316)
Supplement: S1 Table — (DOCX) [file pgph.0004316.s001.docx]

**S1 Table:** Socio-demographic characteristics among ever-pregnant women in Sub-Saharan Africa using the latest DHS 2015-2023 **(**weighted frequency)

| Category | Burkina Faso | Cote’divore | Kenya | Tanzania |
| --- | --- | --- | --- | --- |
| Maternal age |  |  |  |  |
| 15-19 | 621 | 608 | 814 | 1,142 |
| 20-24 | 3622 | 2834 | 5042 | 6,556 |
| 25-29 | 6520 | 5130 | 11112 | 11,721 |
| 30-34 | 9318 | 7389 | 13427 | 13,914 |
| 35-39 | 10579 | 8002 | 16028 | 16,637 |
| 40-44 | 9647 | 6997 | 12885 | 15,432 |
| 45-49 | 7885 | 4706 | 11547 | 14,972 |
| Maternal education |  |  |  |  |
| No education | 38,652 | 24,119 | 8,245 | 20,051 |
| Primary | 5,190 | 6,910 | 36,363 | 49,608 |
| Secondary | 4,008 | 3,887 | 18,019 | 10,181 |
| Higher | 343 | 749 | 8,227 | 533 |
| Partners’ Education |  |  |  |  |
| No education | 37,148 | 18,944 | 5,518 | 10,229 |
| Primary education | 4,680 | 5,315 | 24,941 | 43,062 |
| Secondary education | 3,206 | 5,058 | 15,916 | 9,358 |
| Higher education | 706 | 1,791 | 8,652 | 1,377 |
| Media exposure |  |  |  |  |
| No | 31,557 | 14,667 | 27,824 | 44,885 |
| Yes | 16,636 | 20,998 | 43,030 | 35,489 |
| Wealth index |  |  |  |  |
| Poorest | 10,657 | 9,055 | 15,901 | 16,986 |
| Poorer | 10,396 | 8,178 | 14,819 | 16,583 |
| Middle | 10,492 | 7,070 | 13,991 | 16,714 |
| Richer | 9,507 | 6,316 | 13,283 | 15,659 |
| Richest | 7,141 | 5,046 | 12,861 | 14,431 |
| Residence |  |  |  |  |
| Urban | 10,750 | 16,947 | 22,679 | 21,854 |
| Rural | 37,442 | 18,718 | 48,176 | 58,519 |
