# Supplementary material for: Pregnancy loss and its predictors among ever-pregnant women in Sub-Saharan Africa: Multilevel mixed effect negative binomial regression
Source: PLOS Glob Public Health. 2025 Apr 7;5(4):e0004316. doi: 10.1371/journal.pgph.0004316 (PMC11975090; doi:10.1371/journal.pgph.0004316)
Supplement: Attachment — Submitted filename: Comments on the Manuscrip.28.10.2024.docx [file pgph.0004316.s002.docx]

**Reviewers Comments**

**Title: Pregnancy Loss and Its Predictors among Women Who Ever Pregnant in Four Sub-Saharan African Countries: Multilevel Mixed Effect Negative Binomial Regression**

**Topic:** Since this study was just conducted in two countries of East and West Africa countries, why not just state that in the topic?

**Keywords:** My suggestion is that instead of Number of pregnancy loss it should read *“Number of pregnancy losses” as this will emphasise that each instance is a separate event if a woman has had more than one.*

**Abstract:**

- **Background:** The statement in the background section *“Unfortunately, pregnancy loss data are limited and not universal in Sub-Saharan Africa.”* My suggestion is instead of saying its limited, the authors should consider maybe using underreported and inconsistent record keeping. Since the DHSs for the four countries which are nationally representative collects this data that why they are doing further analysis to ensure that they accurately report the correct estimates.
- **Methods section:** It will be better to indicate even here whether sterile and infecund women were removed from the analysis.
- **Results:** The first sentence should just end after the CI in the brackets, delete “per 10 pregnancies.” Second sentence it should read one year **increase** and not increasing, higher number of household members replace your sentence with this one. Higher number **of** under-five children.
- **Conclusion:** **increases** and not increase the incidence risk of pregnancy loss. The statement that follows, “Mothers with high fertility like having a high number of children reduce pregnancy loss.” Remember there is a difference between TFR, Number of living Children, Children Ever Born etc, be more specific.

**Introduction**

Line 57: You say study when you have two sources “revise to studies.”

Line 60: remove outcomes after still births.

Line 61: there is a word missing between intervals and prevent, please revise so that the sentence is grammatically correct. Equally after studies it should be shows that and not showed

Line 63: Remove also and add based after conducted

Line 74: **of** is missing between number and pregnancy

Line 75 – 80. This is talking about the data source and should not be here but under methodology section.

Line 76: add **the** before number and remove **who** before ever

Lines 51 – 80: Your paragraph is way too long, ensure that you revise so that subject matter components are separated. For example, you have combined factors associated with pregnancy loss, same paragraph is talking about inconsistent data and misreporting whereas the last part is looking at the justification as to why the study is relevant.

*Overall comments for the introduction section:* Though the first paragraph has highlighted the magnitude of pregnancy losses in SSA, and some of the determinants have been highlighted in the first part of the second paragraph, little has been provided on what is happening the four selected SSA countries with what effects are being made regard to pregnancy loss (still birth) data, what the gaps are and why these four countries warrant being studied. The justification as to why these four countries and why the study is being conducted is not clear from the second paragraph.

**Methodology**

Lines 83 – 88: Study design, and study settings and population should be combined and the heading should just be study design. A lot of repetition in the writeup. Like I have highlighted under the introduction section, there is no justifications as to why the four countries were selected for this study? Why are they a focus for this study? Important

Linse 89 – 96: This should be combined into one paragraph and should just read study population only. Under the exclusion criteria, they should remove in their analysis women who were sterile or infecund, without which, the estimates are being underreported as the denominator is having women who are not supposed to be included.

Lines 99 -110: why are you using was and not is, remember the DHS are ongoing surveys

Sample size: since the sample is a pooled one, you need indicate in the Statistical analysis how the sample equalisation factor was applied to avoid results being skewed towards countries with bigger samples.

Line 109: the authors state that the included recent surveys between 2015 – 2023, however, if you check in Figure 1, they state from 2017 – 2023. Kindly revise for consistence’s sake. Equally, it will make senesce in the diagram and in-text you indicate what each country total uncleaned and cleaned samples were

Lines 113-116: My understanding is that, the sampling method should come earlier before the sample size rearrange, please.

Lines 117-118: revise the sentence so that grammatically it reads correct please.

Line 216 – 218: Here you mention that the authorization letter was requested to download the DHS dataset which was obtained from the CSA, are all statistical agencies for the four countries called that? Or you had requested the DHS program to download the four datasets from its website? Kindly clarify and ensure that you amend this component to reflect the true picture please.

**Results**

Include the total sample in Table 1 Frequency (N = XXXX)

Line 225: Just present the percentage. People will check the absolute numbers in the tables.

Line 234: Ensure that the survey period is corrected either 2015 – 2023 or 2017 – 3023

Table: It read partners’ and not partner

Line 236: *were* is missing between who and ever.

Table 3: Ensure that you keep the decimal places for Variance, AIC and BIC the same. The values for the AIC and BIC don’t seem to be rightly written for example under model 0 is it 29,6074 or 290,074 or 29,607.4?

Line 264: You have said LLR symbol when in Table 3 this is not indicated either you remove it here or include it in Table 3.

Line 278: Change all the was to is in this sentence please.

Line 283 – 284: The incidence risk ratio of pregnancy loss was high among women with a higher number of household members should read as “The incidence risk ratio of pregnancy loss was high among women from large households or households with more household members.”

Line 290: Ensure that the reference period is rectified.

**Discussion**

Line 305: as much as you have highlighted the overall for all the four countries used, compare the highest and lowest for the four countries as well.

Lines 309 – 311: This finding highlights the need for better maternal healthcare in these four Sub-Saharan African countries. This statement in its current form does not in anyway provide much value kindly argue why? Just by stating the prevalence and compared to one SSA and two DCs doesn’t warrant that the point has been sufficiently discussed.

Line 311: The sentence starting with the study..... should have its own paragraph because the subject matter changes.

Line 316-319: The points being used to argue your points should be swapped for a simple reason that the last is more closely linked to the discussion, as much as the one used first is its an assertion being used to qualify the point but beyond the scope of this study.

Line 320 – 349: split the paragraphs specific to the point being argued. Its too long and talking about so many different discussion points.

**Strength and limitation:** In your strengths, include that these are nationally representative as they provide better estimates even if they are done every five years

**Conclusion:** A lot of factors have been highlighted as to why there is high pregnancy loss. In your conclusion my take is you should also highlight the importance of delayed age at marriage instead of just age.
