# Supplementary material for: Pregnancy loss and its predictors among ever-pregnant women in Sub-Saharan Africa: Multilevel mixed effect negative binomial regression
Source: PLOS Glob Public Health. 2025 Apr 7;5(4):e0004316. doi: 10.1371/journal.pgph.0004316 (PMC11975090; doi:10.1371/journal.pgph.0004316)
Supplement: Attachment — Submitted filename: Author response to reviewers comment.docx [file pgph.0004316.s003.docx]

**Authors’ Responses to Reviewers Comments**

**Dear Academic Reviewers of PLOS Global Public Health**

“**Pregnancy Loss and Its Predictors among Women Who Ever Pregnant in Four Sub-Saharan African Countries: Multilevel Mixed Effect Negative Binomial Regression**”

Dear PLOS Global Public Health Reviewers;

We are thankful for your constructive comments. We have looked at the comments and have revised our paper accordingly. We hope our paper improved as a result of incorporating the reviewer’s and academic editor’s comments and suggestions. Here are the authors’ responses to the comments.

Please find for your kind consideration the following:

1. Revised manuscript without highlight
2. A revised paper with highlight
3. A rebuttal letter that responds to each point raised by the academic reviewer.

The point-by-point responses of authors are written by hoping these changes would meet with your favorable consideration, we are happy to hear if there are more comments and suggestions. Please do not hesitate to let us know if you have any questions.

Yours Sincerely

Mr. Abel Endawkie Correspondence Author

Department of Epidemiology and Biostatistics School of Public Health College of Medicine and Health Science Wollo University Dessie Ethiopia

Tel. 251935459310	Email address: abelendawkie@gmail.com

We have tried our best to improve it accordingly:

**Point-by-point response of Authors for editor's and reviewer's comment**

Journal Requirements:
1. Thank you for uploading your study's underlying data set. Unfortunately, the repository you have noted in your Data Availability statement does not qualify as an acceptable data repository according to PLOS's standards.
 At this time, please upload the minimal data set necessary to replicate your study's findings to a stable, public repository (such as figshare or Dryad) and provide us with the relevant URLs, DOIs, or accession numbers that may be used to access these data. For a list of recommended repositories and additional information on PLOS standards for data deposition, please see <https://journals.plos.org/plosone/s/recommended-repositories>.

**Response 1**: I thank you and appreciate your prompt and timely response. To ensure compliance with PLOS Global Public Health formatting guidelines, we have meticulously followed the typesetting requirements for references, tables, and technical specifications for figures and to expedite the process and prevent any delays.

2. Please provide an Author Summary. This should appear in your manuscript between the Abstract (if applicable) and the Introduction, and should be 150–200 words long. The aim should be to make your findings accessible to a wide audience that includes both scientists and non-scientists. Sample summaries can be found on our website under Submission Guidelines: <https://journals.plos.org/globalpublichealth/s/submission-guidelines#loc-parts-of-a-submission>

**Response 2:** I thank you and appreciate your prompt and timely response. To ensure compliance with PLOS Global Public Health formatting guidelines, we have meticulously followed the typesetting requirements for references, tables, and technical specifications for figures and to expedite the process and prevent any delays.

Additional Editor Comments (if provided):
Reviewers' comments:

**Title: Pregnancy Loss and Its Predictors among Women Who Ever Pregnant in Four Sub-Saharan African Countries: Multilevel Mixed Effect Negative Binomial Regression**

**Comment 1: Topic:** Since this study was just conducted in two countries of East and West Africa countries, why not just state that in the topic?

Response 1: Thank you for your valuable comment and suggestion. I apologize for any confusion in our writing. In my review of the literature, I noted that between 2015 and 2020, Africa had an average fertility rate of 4.44 children per woman, significantly higher than the global average of 2.47. Despite this high fertility rate, pregnancy loss remains a critical maternal health issue across Sub-Saharan Africa. According to the World Health Organization (WHO), the continent has one of the highest stillbirth rates globally, with an estimated 2.7 million stillbirths occurring each year. This highlights the profound impact of pregnancy loss on families throughout the region.

My interest in this topic stems from a desire to understand the factors contributing to the numbers of pregnancy loss in Sub-Saharan Africa. While I explore the data to conduct this research, I found that only four countries in Sub-Saharan Africa had information on pregnancy loss (including spontaneous abortion/miscarriage, induced abortion, and stillbirth) on their the latest DHS data available from 2015-2023, two of which are located in East Africa and two in West Africa. Therefore, I chose to frame the study within the broader context of Sub-Saharan Africa to encompass these findings comprehensively, rather than focusing solely on individual countries. For comprehensive understanding, I had revised the title, as follow “**Number of Pregnancy Loss and Its Predictors among Ever Pregnant Women in Sub-Saharan Africa: Multilevel Mixed Effect Negative Binomial Regression”** Thank you for your understanding, and I appreciate your feedback on clarifying the study's scope.

**Comment 2: Keywords:** My suggestion is that instead of Number of pregnancy loss it should read “Number of pregnancy losses” as this will emphasise that each instance is a separate event if a woman has had more than one.

Response 2: Thank you for your valuable feedback! I appreciate your suggestion and have made the necessary amendment to reflect "Number of pregnancy losses" to highlight each instance as a distinct event. I apologize for any confusion caused by the previous wording.

**Abstract:**

**Comment 3: Background:** The statement in the background section *“Unfortunately, pregnancy loss data are limited and not universal in Sub-Saharan Africa.”* My suggestion is instead of saying its limited, the authors should consider maybe using underreported and inconsistent record keeping. Since the DHSs for the four countries which are nationally representative collects this data that why they are doing further analysis to ensure that they accurately report the correct estimates.

**Response 3:** Thank you for your insightful comment. I appreciate your thorough review and suggestion. I agree that using terms like "underreported" and "inconsistent record keeping" more accurately reflects the situation regarding pregnancy loss data in Sub-Saharan Africa. I have revised the background section accordingly to clarify this point and emphasize the importance of the DHS data from the four countries, which supports our further analysis for accurate estimates. Thank you for your valuable input!

**Comment 4: Methods section:** It will be better to indicate even here whether sterile and infecund women were removed from the analysis.

Response 4: Thank you for your insightful comment regarding the methods section. I appreciate your thorough review of the content. I apologize for any confusion caused by my previous writing. I have made revisions to clarify whether sterile and infecund women were excluded from the analysis. Your feedback has been invaluable in enhancing the clarity of the study. Thank you once again for your attention to detail.

**Comment 5: Results:** The first sentence should just end after the CI in the brackets, delete “per 10 pregnancies.” Second sentence it should read one year increase and not increasing, higher number of household members replace your sentence with this one. Higher number of under-five children.

Response 5: Thank you for your thoughtful feedback. I appreciate your careful review of the content. I apologize for any confusion caused by the phrasing. I have made the necessary revisions, including ending with CI, and adjusting the wording to "one year increase," and replacing the sentence with "higher number of under-five children." Thank you for your valuable suggestions!

Comment 6: **Conclusion:** **increases** and not increase the incidence risk of pregnancy loss. The statement that follows, “Mothers with high fertility like having a high number of children reduce pregnancy loss.” Remember there is a difference between TFR, Number of living Children, Children Ever Born etc, be more specific.

Response 6: Thank you for your thoughtful comment regarding the conclusion. I appreciate your careful review of the content. I apologize for any confusion caused by our wording. I have revised the statement to clarify the distinctions between Total Fertility Rate (TFR), number of living children, and children ever born, ensuring greater specificity in our conclusions. Thank you once again for your valuable feedback.

**Introduction**

**Comment 7:** Line 57: You say study when you have two sources “revise to studies.

Response 7: Thank you for your insightful comment. I appreciate your careful review of the content. I apologize for any confusion caused by my wording. I have revised the text to use "studies" in place of "study." Thank you for bringing this to our attention.

Comment 8: Line 60: remove outcomes after still births.

Response 8: I would like to thank you for your deep comment. I appreciated your efforts in reviewing the content. I would like to apologize for we confused you in writing. I had revised and corrected it.

Comment 9: Line 61: there is a word missing between intervals and prevent, please revise so that the sentence is grammatically correct. Equally after studies it should be shows that and not showed.

Response 9: I would like to thank you for your deep comment. I appreciated your efforts in reviewing the content. I would like to apologize for we confused you in writing. I had revised and corrected it.

Comment 10: Line 63: Remove also and add based after conducted

Response 10: I would like to thank you for your deep comment. I appreciated your efforts in reviewing the content. I would like to apologize for we confused you in writing. I had revised and corrected it.

Comment 11: Line 74: **of** is missing between number and pregnancy

Response 11: Thank you for your insightful comment. I appreciate the time you took to review the content. I apologize for any confusion caused by the writing. I have made the necessary revisions to correct the issue.

Comment 12: Line 75 – 80. This is talking about the data source and should not be here but under methodology section.

Response 12: I would like to thank you for your deep comment. I appreciated your efforts in reviewing the content. I would like to apologize for we confused you in writing. I had revised and corrected it.

Comment 13: Line 76: add **the** before number and remove **who** before ever

Response 13: I would like to thank you for your deep comment. I appreciated your efforts in reviewing the content. I would like to apologize for we confused you in writing. I had revised and corrected it.

Comment 14: Lines 51 – 80: Your paragraph is way too long, ensure that you revise so that subject matter components are separated. For example, you have combined factors associated with pregnancy loss, same paragraph is talking about inconsistent data and misreporting whereas the last part is looking at the justification as to why the study is relevant.

*Overall comments for the introduction section:* Though the first paragraph has highlighted the magnitude of pregnancy losses in SSA, and some of the determinants have been highlighted in the first part of the second paragraph, little has been provided on what is happening the four selected SSA countries with what effects are being made regard to pregnancy loss (still birth) data, what the gaps are and why these four countries warrant being studied. The justification as to why these four countries and why the study is being conducted is not clear from the second paragraph.

Response 14: Thank you for your valuable feedback. I appreciate your thorough review of the content. I understand the concerns regarding the lengthy paragraph and the mixing of different subject matters. I have revised to clarify inconsistent of previous study finding, and the study's relevance. Additionally, I tried to enhance the introduction by providing more specific information about the situation in the SSA countries rather than at these specific countries, including the gaps in the evidence and the rationale for conducting this study. I have revised the components related to pregnancy loss data and inserted in method section. Your suggestions is valuable and will help clarify the justification for this research, and I had tried to ensure these revisions are made change your expectation.

**Here is why I only select these countries for this analysis.** I interested to do this research. My interest in this topic stems from a desire to understand the factors contributing to the prevalence of pregnancy loss in Sub-Saharan Africa. While conducting my research, I found that only four countries in Sub-Saharan Africa had information on pregnancy loss (including spontaneous abortion/miscarriage, induced abortion, and stillbirth on the latest Demographic and Health Survey (DHS) data available from 2015-2023, two of which are located in East Africa and two in West Africa. Therefore, I chose to frame the study within the broader context of Sub-Saharan Africa to encompass these findings comprehensively, rather than focusing solely on individual countries. Thank you for your understanding, and I appreciate your feedback on clarifying the study's scope. Therefore, this study included Burkina Faso, Côte d'Ivoire, Kenya, and Tanzania, because of their latest demographic and health survey (DHS) data which had information on pregnancy loss (including spontaneous abortion/miscarriage, induced abortion, and stillbirth) available during this period. Countries lacking information on pregnancy loss (including spontaneous abortion/miscarriage, induced abortion, and stillbirth) on their latest DHS data were excluded from the analysis.

**The reason why I selected DHS period from 2015-2023 is highlighted below.**

In my review of the literature, I noted that between 2015 and 2020, Africa had an average fertility rate of 4.44 children per woman, significantly higher than the global average of 2.47. Despite this high fertility rate, pregnancy loss remains a critical maternal health issue across Sub-Saharan Africa. According to the World Health Organization (WHO), the continent has one of the highest stillbirth rates globally, with an estimated 2.7 million stillbirths occurring each year. This highlights the profound impact of pregnancy loss on families throughout the region and called what factor contribute for this problem in this period.

**Dear, reviewer I hope you will understand my interest and work.**

**Methodology**

Comment 15: Lines 83 – 88: Study design, and study settings and population should be combined and the heading should just be study design. A lot of repetition in the write up. Like I have highlighted under the introduction section, there is no justifications as to why the four countries were selected for this study? Why are they a focus for this study? Important

Response 15: I would like to thank you for your deep comment. I appreciated your efforts in reviewing the content. I would like to apologize for we confused you in writing. I had revised and corrected it.

Comment 16: Line 89 – 96: This should be combined into one paragraph and should just read study population only. Under the exclusion criteria, they should remove in their analysis women who were sterile or infecund, without which, the estimates are being underreported as the denominator is having women who are not supposed to be included.

Response 16: I would like to thank you for your deep comment. I appreciated your efforts in reviewing the content. I would like to apologize for we confused you in writing. I had revised and corrected it.

Comment 17: Lines 99 -110: why are you using was and not is, remember the DHS are ongoing surveys. Sample size: since the sample is a pooled one, you need indicate in the Statistical analysis how the sample equalisation factor was applied to avoid results being skewed towards countries with bigger samples.

Response 17: I would like to thank you for your deep comment. I appreciated your efforts in reviewing the content. I would like to apologize for we confused you in writing. I had revised and corrected it.

Comment 18: Line 109: the authors state that the included recent surveys between 2015 – 2023, however, if you check in Figure 1, they state from 2017 – 2023. Kindly revise for consistence’s sake. Equally, it will make senesce in the diagram and in-text you indicate what each country total uncleaned and cleaned samples were.

Response 18: Thank you for your insightful comment. I appreciate your careful review of the content. I apologize for the inconsistency regarding the survey dates and any confusion it may have caused. I have revised the text to ensure that the time frame is consistently stated as 2015–2023 throughout. Additionally, I have updated Figure 1 to include the total uncleaned and cleaned samples for each country, as you suggested. Your feedback is invaluable, and I have made these corrections accordingly.

Comment 19: Lines 113-116: My understanding is that, the sampling method should come earlier before the sample size rearrange, please.

Response 19: I would like to thank you for your deep comment. I appreciated your efforts in reviewing the content. I would like to apologize for we confused you in writing. I had revised and rearranged it.

Comment 20: Lines 117-118: revise the sentence so that grammatically it reads correct please.

Response 20: I would like to thank you for your deep comment. I appreciated your efforts in reviewing the content. I would like to apologize for we confused you in writing. I had revised and corrected it.

Comment 21: Line 216 – 218: Here you mention that the authorization letter was requested to download the DHS dataset which was obtained from the CSA, are all statistical agencies for the four countries called that? Or you had requested the DHS program to download the four datasets from its website? Kindly clarify and ensure that you amend this component to reflect the true picture please.

Response 21: Thank you for your insightful comment. I appreciate your careful review of the content. I apologize for any confusion caused by my wording. To clarify, I submitted a single authorization letter to the CSA to request access to the DHS datasets, which encompasses all DHS dataset of SSA countries rather than submitting separate requests for each countries. I have revised the text to accurately reflect this information, as you suggested. Thank you for your guidance.

**Results**

Comment 22: Include the total sample in Table 1 Frequency (N = XXXX)

Response 22: I would like to thank you for your deep comment. I appreciated your efforts in reviewing the content. I would like to apologize for we confused you in writing. I had revised and corrected it.

Comment 23: Line 225: Just present the percentage. People will check the absolute numbers in the tables.

Response 23: I would like to thank you for your deep comment. I appreciated your efforts in reviewing the content. I would like to apologize for we confused you in writing. I had revised and corrected it.

Comment 24: Line 234: Ensure that the survey period is corrected either 2015 – 2023 or 2017 – 3023

Response 24: I would like to thank you for your deep comment. I appreciated your efforts in reviewing the content. I would like to apologize for we confused you in writing. I had revised and corrected it.

Comment 25: Table: It read partners’ and not partner

Response 25: Thank you for your thoughtful comment. I appreciate your careful review of the content. I apologize for any confusion caused by the wording. I have revised the table to correct "partner" to "partners’." Thank you for bringing this to my attention.

Comment 26: Line 236: *were* is missing between who and ever.

Response 26: I would like to thank you for your deep comment. I appreciated your efforts in reviewing the content. I would like to apologize for we confused you in writing. I had revised and corrected it.

Comment 27: Table 3: Ensure that you keep the decimal places for Variance, AIC and BIC the same. The values for the AIC and BIC don’t seem to be rightly written for example under model 0 is it 29, 6074 or 290,074 or 29,607.4?

Response 78: I would like to thank you for your deep comment. I appreciated your efforts in reviewing the content. I would like to apologize for we confused you in writing. I had revised and corrected it.

Comment 29: Line 264: You have said LLR symbol when in Table 3 this is not indicated either you remove it here or include it in Table 3.

Response 29: I would like to thank you for your deep comment. I appreciated your efforts in reviewing the content. I would like to apologize for we confused you in writing. I had revised and corrected it.

Comment 30: Line 278: Change all the was to is in this sentence please.

Response 30: I would like to thank you for your deep comment. I appreciated your efforts in reviewing the content. I would like to apologize for we confused you in writing. I had revised and corrected it.

Comment 31: Line 283 – 284: The incidence risk ratio of pregnancy loss was high among women with a higher number of household members should read as “The incidence risk ratio of pregnancy loss was high among women from large households or households with more household members.”

Response 31: I would like to thank you for your deep comment. I appreciated your efforts in reviewing the content. I would like to apologize for we confused you in writing. I had revised and corrected it.

Comment 32: Line 290: Ensure that the reference period is rectified.

Response 32: I would like to thank you for your deep comment. I appreciated your efforts in reviewing the content. I would like to apologize for we confused you in writing. I had revised and corrected it.

**Discussion**

Comment 33: Line 305: as much as you have highlighted the overall for all the four countries used, compare the highest and lowest for the four countries as well.

Response 33: I would like to thank you for your deep comment. I appreciated your efforts in reviewing the content. I would like to apologize for we confused you in writing. I had revised and corrected it.

Comment 34: Lines 309 – 311: This finding highlights the need for better maternal healthcare in these four Sub-Saharan African countries. This statement in its current form does not in anyway provide much value kindly argue why? Just by stating the prevalence and compared to one SSA and two DCs doesn’t warrant that the point has been sufficiently discussed.

Response 34: I would like to thank you for your deep comment. I appreciated your efforts in reviewing the content. I would like to apologize for we confused you in writing. I had revised and corrected it. Here is the revised paragraph. This study finding is higher than the mean number of pregnancy loss reported in the United States (1 pregnancy loss per 10 pregnancies) (10), Manitoba (1 pregnancy loss per 10 pregnancies) (29), and the United Kingdom (1 pregnancy loss per 10 pregnancies) (30). This highlights a significant public health issue regarding pregnancy loss in Sub-Saharan Africa compared to more developed regions. The disparity in the rates of pregnancy loss may due to limited access to healthcare, socioeconomic disparities, and cultural practices that may affect maternal health, lack of access to skilled healthcare providers, and insufficient maternal health education in Sub-Saharan Africa. This finding highlights the need for better maternal healthcare in Sub-Saharan Africa.

Comment 35: Line 311: The sentence starting with the study..... should have its own paragraph because the subject matter changes.

Response 35: I would like to thank you for your deep comment. I appreciated your efforts in reviewing the content. I would like to apologize for we confused you in writing. I had revised and corrected it.

Comment 36: Line 316-319: The points being used to argue your points should be swapped for a simple reason that the last is more closely linked to the discussion, as much as the one used first is its an assertion being used to qualify the point but beyond the scope of this study.

Response 36: I would like to thank you for your deep comment. I appreciated your efforts in reviewing the content. I would like to apologize for we confused you in writing. I had revised and corrected it.

Comment 37: Line 320 – 349: split the paragraphs specific to the point being argued. Its too long and talking about so many different discussion points.

Response 37: I would like to thank you for your deep comment. I appreciated your efforts in reviewing the content. I would like to apologize for we confused you in writing. I had revised and corrected it.

**Comment 38: Strength and limitation:** In your strengths, include that these are nationally representative as they provide better estimates even if they are done every five years

Response 38: I would like to thank you for your deep comment. I appreciated your efforts in reviewing the content. I would like to apologize for we confused you in writing. I had revised and corrected it.

**Comment 39: Conclusion:** A lot of factors have been highlighted as to why there is high pregnancy loss. In your conclusion my take is you should also highlight the importance of delayed age at marriage instead of just age.

Response 39: I would like to thank you for your deep comment. I appreciated your efforts in reviewing the content. I would like to apologize for we confused you in writing. I had revised and corrected it.

**Finally, I appreciate your kind response and genuine communication. I hope these changes will meet your favourable consideration and I am happy to hear if there are more comments and suggestions. Please do not hesitate to let me know if you have any questions.**

**I feel all of you may understand our feelings and interests.**

**I am thankful for all!!**
