# Supplementary material for: Pregnancy loss and its predictors among ever-pregnant women in Sub-Saharan Africa: Multilevel mixed effect negative binomial regression
Source: PLOS Glob Public Health. 2025 Apr 7;5(4):e0004316. doi: 10.1371/journal.pgph.0004316 (PMC11975090; doi:10.1371/journal.pgph.0004316)
Supplement: Attachment — Submitted filename: Author_response_to_reviewers_comment_auresp_2.docx [file pgph.0004316.s004.docx]

**Authors’ Responses to Reviewers Comments**

**Dear Academic Reviewers of PLOS Global Public Health**

“**Number of Pregnancy Loss and Its Predictors among Ever Pregnant Women in Sub-Saharan Africa: Multilevel Mixed Effect Negative Binomial Regression”**

Dear PLOS Global Public Health Reviewers;

We are thankful for your constructive comments. We have looked at the comments and have revised our paper accordingly. We hope our paper improved as a result of incorporating the reviewer’s and academic editor’s comments and suggestions. Here are the authors’ responses to the comments.

Please find for your kind consideration the following:

1. Revised manuscript without highlight
2. A revised paper with highlight
3. A rebuttal letter that responds to each point raised by the academic reviewer.

The point-by-point responses of authors are written by hoping these changes would meet with your favorable consideration, we are happy to hear if there are more comments and suggestions. Please do not hesitate to let us know if you have any questions.

Yours Sincerely

Mr. Abel Endawkie Correspondence Author

Department of Epidemiology and Biostatistics School of Public Health College of Medicine and Health Science Wollo University Dessie Ethiopia

Tel. 251935459310	Email address: abelendawkie@gmail.com

We have tried our best to improve it accordingly:

**Point-by-point response of Authors for editor's and reviewer's comment**

Journal Requirements:
1. Thank you for uploading your study's underlying data set. Unfortunately, the repository you have noted in your Data Availability statement does not qualify as an acceptable data repository according to PLOS's standards.
 At this time, please upload the minimal data set necessary to replicate your study's findings to a stable, public repository (such as figshare or Dryad) and provide us with the relevant URLs, DOIs, or accession numbers that may be used to access these data. For a list of recommended repositories and additional information on PLOS standards for data deposition, please see <https://journals.plos.org/plosone/s/recommended-repositories>.

**Response 1**: I thank you and appreciate your prompt and timely response. To ensure compliance with PLOS Global Public Health formatting guidelines, we have meticulously followed the typesetting requirements for references, tables, and technical specifications for figures and to expedite the process and prevent any delays.

**Reviewer comment**

General comment: I have read the manuscript by Endawkie et al. titled “Number of Pregnancy Loss and Its Predictors among Ever Pregnant Women in Sub-Saharan Africa: Multilevel Mixed-Effect Negative Binomial Regression” with great interest. I agree with the authors that understanding the underlying causes of pregnancy loss is crucial for reducing uneventful pregnancies, which often bring significant social stigma to families and increase their vulnerability. Sub-Saharan Africa, in particular, lags behind other regions in addressing this issue, underscoring the importance of this study.

I have also reviewed how the authors addressed the flagged areas raised by previous reviewers. While the current version of the manuscript resolves most of these concerns, there are still some areas requiring improvement before it is ready for publication in PLOS Global Public Health Journal. Below are my specific comments and suggestions for revisions:

**Response:** Dear reviewer, thank you so much for your kind words about our work! We truly appreciate your thoughtful feedback. We've carefully considered your recommendations and made revisions to enhance our manuscript. Your insights have been invaluable, and we look forward to your thoughts on the changes and hoping these changes would meet with your favorable consideration. Thank you again for your encouraging and positive feedback.

Abstract

• Line 20: Please specify whether the value 0.267 represents the mean pregnancy loss per woman or the proportion of pregnancy loss.

**Response 1:** Thank you for your insightful question. To clarify, the value of 0.267 represents the average number of pregnancy losses per woman, rather than a proportion or prevalence. We employed a count model that accounts for the total and average number of pregnancy losses experienced by women, specifically using multilevel negative binomial regression, which is an extension of Poisson regression. In our analysis, we did not categorize the records; instead, we treated the dependent variable as the total number of pregnancy losses that each woman had experienced prior to the survey. This was recorded as (0, 1, 2, 3, 4, 5, 6… 9), with a minimum of 0 and a maximum of 9. Thank you for your understanding. Look line #120-121 and table 2.

• Line 23: Clarify what is meant by "a higher number of under-five children." Does this refer to a higher count of children under five per household or something else?

**Response 2:** Dear Reviewer, thank you for your valuable feedback. To clarify, "a higher number of under-five children" indeed refers to a greater count of children under five years of age per household.

• Lines 26–32: The conclusion is somewhat repetitive of the results section. Consider providing a concise take-home message with actionable steps or policy recommendations to reduce pregnancy loss risk in sub-Saharan Africa (SSA).

**Response 3:** Thank you for your genuine communication and scientific suggestion. We had revised the conclusion which make sensible and actionable the maximum effort. Refer line# 31-40

Introduction

• Line 68: The phrase "information loss" is ambiguous. Does this refer to a lack of data on pregnancy loss? Please clarify.

**Response 4:** Thank you for your valuable feedback. We had revised to clarify. We mean that, categorizing women who have several pregnancy losses in the same group and the repeated pregnancy loss may lead information loss and is more likely to be intolerable than single pregnancy loss for families and mothers are especially hurting by the number of losses and they commonly have broken heart for repeated loss of pregnancy. Therefore conducting a count data analysis is important to account the total loss rather than categorizing in one category as no and yes. Please the see revised version.

Methods

• Line 79: The sentence implies that the authors conducted the survey themselves. Please revise so readers understand that DHS data was used.

**Response 5:** Thank you for your scholarly suggestion and comment. We had revised and corrected it. See line # 87

• Line 92: The term "viable birth" may need clarification. Consider replacing it with pregnancy outcome for greater accuracy.

**Response 6:** Thank you for your scholarly suggestion and comment. We had revised and corrected it. See line # 101.

Results

• Line 268: The authors express the value in percentages here, while using proportion in other sections. Please consider using a consistent format throughout for better readability.

**Response 7:** Thank you for your kind words and thoughtful feedback. We would like to clarify that, the percentage in our result indicated only the socio-demographic characteristics respondents. For example from the total number of ever pregnant women, 91,068 (38.7%) were not educated.

In the case of line 268, we would like to clarify that, the percentage calculation to interpreate the adjusted incident rate ratio. The percentage increase in the risk of pregnancy loss can be derived from the Adjusted Incidence Rate Ratio (AIRR). Here's how we are interpret and calculate it:

For example: understanding the AIRR: The AIRR of 1.067 indicates that for each additional year of maternal age, the rate of pregnancy loss increases by 6.7%.

The formula to calculate the percentage increase from an incidence rate ratio (IRR) is: Percentage Increase = (IRR - 1) × 100. Therefore, maternal age, percentage increase = (1.067 - 1) × 100 = 0.067 × 100 = 6.7%. This is what we did.

• Table 4: Including a p-value for the adjusted incidence rate ratio (AIRR) would facilitate easier interpretation.

**Response 8:** Thank you for your scientific recommendation. However we have provided the Confidence Interval (95% CI) which directly tell us the message of p-value. The CI interval which is not included null value means no included 1 is statically significant. For example, the confidence interval provided is (1.06, 1.07). This range suggests that we can be 95% confident that the true value of the AIRR lies between 1.06 and 1.07. Since the entire interval is above 1, it reinforces the conclusion that increasing maternal age is associated with a higher risk of pregnancy loss. So writing the p-value may important when the confidence interval is not reported unless it is redundancy. As we know confidence interval is more informative than p-value in statistics. Some of reason.

1. Range of Values: A confidence interval (CI) provides a range of values within which we can be reasonably confident that the true parameter (e.g., mean, proportion) lies. This range gives context to the estimate and allows researchers to understand the potential variability in their data. In contrast, a p-value only indicates whether an effect exists (e.g., whether to reject the null hypothesis) but does not provide information about the magnitude or direction of that effect.

2. Magnitude and Precision: Confidence intervals convey not only whether an effect is statistically significant but also how large that effect might be. For example, a CI that ranges from 1.06 to 1.07 suggests a meaningful effect, whereas a p-value of 0.05 could result from a very small effect size. Knowing the CI helps researchers assess the precision of their estimates, as narrower intervals indicate greater precision.

• A supplementary table showing the population characteristics by country would enhance the manuscript, as country-specific variations in population characteristics could influence the overall outcomes.

**Response 9:**

Discussion

• Line 293: The format of presenting values (e.g., "3 in 10 pregnancies") is inconsistent with other sections where mean numbers are used. Please adopt a single format (either proportion or percentage) throughout the manuscript.

**Response 10:** Thank you for your insightful comment. To clarify, the value of 0.267 represents the average number of pregnancy losses per woman, rather than a proportion or prevalence. We employed a count model that accounts for the total and average number of pregnancy losses experienced by women, specifically using multilevel negative binomial regression, which is an extension of Poisson regression. In our analysis, we did not categorize the records; instead, we treated the dependent variable as the total number of pregnancy losses that each woman had experienced prior to the survey. This was recorded as (0, 1, 2, 3, 4, 5, 6… 9), with a minimum of 0 and a maximum of 9. Therefore it is mean throughout. please see table 2. Thank you for your understanding.

• Line 304: The statement about mean pregnancy loss in Burkina Faso being relatively higher is unclear. Specify what it is being compared to and include appropriate citations.

**Response 11:** Thank for your concern and we appreciated you’re deeply insight. We have revised and corrected it.

• Line 315: The claim that primary education is linked with increased pregnancy loss is not supported by the data presented in Table 4. Please provide additional evidence or clarify this point.

**Response 12:** Thank for your concern and we appreciated you’re deeply insight. Please look table 4 row r column 5. Read as” 1.10(1.01, 1.22)*”

**Finally, I appreciate your kind response and genuine communication. I hope these changes will meet your favourable consideration and I am happy to hear if there are more comments and suggestions. Please do not hesitate to let me know if you have any questions.**

**I feel all of you may understand our feelings and interests.**

**I am thankful for all!!**
