# Supplementary material for: Pregnancy loss and its predictors among ever-pregnant women in Sub-Saharan Africa: Multilevel mixed effect negative binomial regression
Source: PLOS Glob Public Health. 2025 Apr 7;5(4):e0004316. doi: 10.1371/journal.pgph.0004316 (PMC11975090; doi:10.1371/journal.pgph.0004316)
Supplement: Attachment — Submitted filename: Author_response_to_reviewers_comment_auresp_3.docx [file pgph.0004316.s005.docx]

**Authors’ Responses to Reviewers Comments**

**Dear Academic Reviewers of PLOS Global Public Health**

“**Number of Pregnancy Loss and Its Predictors among Ever Pregnant Women in Sub-Saharan Africa: Multilevel Mixed Effect Negative Binomial Regression”**

Dear PLOS Global Public Health Reviewers;

We are thankful for your constructive comments. We have looked at the comments and have revised our paper accordingly. We hope our paper improved as a result of incorporating the reviewer’s and academic editor’s comments and suggestions. Here are the authors’ responses to the comments.

Please find for your kind consideration the following:

1. Revised manuscript without highlight
2. A revised paper with highlight
3. A rebuttal letter that responds to each point raised by the academic reviewer.

The point-by-point responses of authors are written by hoping these changes would meet with your favorable consideration, we are happy to hear if there are more comments and suggestions. Please do not hesitate to let us know if you have any questions.

Yours Sincerely

Mr. Abel Endawkie Correspondence Author

Department of Epidemiology and Biostatistics School of Public Health College of Medicine and Health Science Wollo University Dessie Ethiopia

Tel. 251935459310	Email address: abelendawkie@gmail.com

We have tried our best to improve it accordingly:

**Point-by-point response of Authors for editor's and reviewer's comment**

**Editor comment:** Thank you for sharing the revised version of the manuscript titled “Number of Pregnancy Loss and Its Predictors among Ever Pregnant Women in Sub-Saharan Africa: Multilevel Mixed-Effect Negative Binomial Regression.” The revisions have enhanced the clarity regarding the study’s methodology and findings. However, I still have a few concerns:

**Response:** Dear editor, thank you so much for your kind words about our work! We truly appreciate your thoughtful feedback. We've carefully considered your recommendations and made necessary revisions to enhance our manuscript. Your insights have been invaluable, and we look forward to your thoughts on the changes and hoping these changes would meet with your favorable consideration. Thank you again for your encouraging and positive feedback. For better understanding we revised the title “Pregnancy Loss and Its Predictors among Ever Pregnant Women in Sub-Saharan Africa: Multilevel Mixed Effect Negative Binomial Regression”.

Comment 1: Line 25: The value 0.267 does not fully align with the explanation provided in the feedback. In the results section, lines 240-242, the authors reported “average number of pregnancy losses among ever-pregnant women was 2.67±0.63 per 10 pregnancies.” Hence, the value 0.267 reflects a proportion rather than the mean number of pregnancy losses per woman. Please clarify.

**Response 1:** Thank you for your insightful question. We really sorry for making you confused in our reporting. Actually 2.67 is not proportion and 0.267 is miss and inconsistence reporting after analysis. Our analysis focused on the number of pregnancy losses per woman, rather than a proportion or prevalence at the population level and the actual figure/ represents mean number and it is no proportion. We employed a count model to determine predictors that accounts for the number of pregnancy losses experienced by women, specifically using multilevel negative binomial regression, which is an extension of Poisson regression. We are really sorry again for making you confused in writing. In our analysis, we did not categorize the records; instead, we treated the dependent variable as the total number of pregnancy losses that each woman had experienced prior to the survey which was recorded as (0, 1, 2, 3, 4, 5, 6… 9), with a minimum of 0 and a maximum of 9. We re-explore the analysis and the actual result and our report is inconsistent. The correct result showed that the number of pregnancy loss is 2.67, 95%CI (2.64, 2.69) with 0.63 standard division. We really appreciate you concern and we made necessary amendment. Thank you for your genuine concern and understanding. Look table 1 and 2.

Comment 2: I appreciate the effort to clarify the term "information loss" in Line 76, but the statement remains unclear. I understand the authors' point that treating women with multiple pregnancy losses the same as those with a single loss may obscure important distinctions. I suggest simplifying this phrasing to enhance readability.

Response 2: Thank you for your insightful feedback. We apologize for any confusion caused by our wording. We have revised the phrasing to make it clearer and more accessible for readers.

Comment 3: The title “Mean numbers of pregnancy loss among ever-pregnant women" (line 239) seems inappropriate as the data presented in the manuscript is expressed as a proportion. Additionally, interpreting findings based on the average number of pregnancy losses per woman does not make sense, as it is expected that older women will have a higher mean number of pregnancy losses than younger women, since younger women have experienced fewer pregnancies and therefore face less risk of pregnancy loss.

Response 3: Thank you for your insightful comment regarding the title of our manuscript. For simple understand we have revised the title to “Pregnancy Loss and Its Predictors among Ever-Pregnant Women in Sub-Saharan Africa: Multilevel Mixed-Effect Negative Binomial Regression” to better reflection.

We appreciate your observation the data presented in our study is similarly expressed as a proportion rather than a mean however the actual analysis indeed counting of pregnancy loss per women who ever pregnant, and we understand the way of interpreting the average number of pregnancy losses per woman may lead to confusion. However, our focus is to understand the number of pregnancy loss and it predictors among ever pregnant women in SSA using count model. As you pointed out, older women are likely to have experienced more pregnancies and, consequently, a higher mean number of pregnancy losses compared to younger women, who have had fewer pregnancies and thus results a lower risk of loss.

To clarify our findings, we employed a multilevel negative binomial regression model to analyze the predictors of number of pregnancy loss. Our results support your claim and indicate that with each additional year of maternal age, there is an associated increase in the rate of pregnancy loss, specifically an adjusted incidence rate ratio (AIRR) of 1.05 [95% CI (1.06, 1.07)]. This reinforces your point regarding the relationship between age and pregnancy loss. As researcher even your logical reasoning is true we believe pointing out statistically is important for evidence. That is why we consider it. We have made revisions throughout the manuscript to enhance clarity and ensure accurate representation of our findings. Thank you once again for your valuable input.

Comment 4: The manuscript suggests primary education is linked to increased pregnancy loss, while higher education is not. This contradicts existing literature indicating that maternal education generally improves pregnancy outcomes. The conclusion seems to rely on a 95% CI (1.01, 1.22) that narrowly excludes the null. Additionally, data from Table 1 and the univariate analysis in Table 4 do not strongly support this claim. Given the policy implications, I recommend providing stronger evidence or further clarification.

Response 4: Thank you for your deep concern. Our finding reveal women with education have a higher incidence risk of pregnancy loss compared to women with no education. The confidence interval of pairwise mean number of pregnancy loss with education indicated wide uncertainty interval for higher rate than no education. As you said this finding was contradicted by the previous study findings (19, 30, 31). The statistical finding we have presented regarding education status was no expected. However, possibly educational status may linked to delay in marriage and pregnancy and late pregnancy intern increases chromosomal abnormality in fetes which increases pregnancy loss. Dear editor you may understand us, we forward our statistical finding and possible justification as researcher. In other ways women with no education may have less access to healthcare (like induced abortion), which could lead to a lower incidence risk of pregnancy loss and women. However, this finding is not supported by other study findings (17, 18). The logical reason may be due to educated mothers may have delayed childbearing and pregnancy due to waste of their time in education and learning which in turn increases chromosomal abnormality in fetes which increases pregnancy loss.

However, we recognize that this cross-sectional study design limits our ability to draw definitive causal conclusions. We revised the presentation of finding for better understanding. Thank you for emphasizing the importance of this issue, particularly given its policy implications. We have provided flexible recommendation like promoting flexible educational pathways and providing career support can encourage healthier timing for pregnancies.
Comment 5: As mentioned earlier, throughout the manuscript, the data are presented in an inconsistent format. For example, in lines 228-230, it is mentioned that the mean number of pregnancy losses was 0.28 per woman who was not educated, while in lines 240-242, it states that an average of 2.67±0.63 pregnancies were lost per 10 pregnancies.

Response 5: Thank you for pointing out the inconsistency in the data presentation. We have thoroughly reviewed and revised our manuscript to ensure a uniform format throughout. Your feedback is invaluable, and we appreciate your understanding as we work to enhance the clarity of our report.

Comment 6: To improve readability and avoid confusion, I recommend ensuring consistent data presentation throughout the manuscript.

Response 6: Thank you for your valuable suggestion regarding the consistency of data presentation throughout the manuscript. We have made the necessary amendments specially the result section to enhance readability and clarity. To avoid the confusion, we revised table 1 by considering the frequency and percentage to characterize the study population only. We appreciate your understanding and feedback.

**Finally, I appreciate your kind response and genuine communication. I hope these changes will meet your favourable consideration and I am happy to hear if there are more comments and suggestions. Please do not hesitate to let me know if you have any questions.**

**I feel all of you may understand our feelings and interests.**

**I am thankful for all!!**
