# Supplementary material for: Pregnancy loss and its predictors among ever-pregnant women in Sub-Saharan Africa: Multilevel mixed effect negative binomial regression
Source: PLOS Glob Public Health. 2025 Apr 7;5(4):e0004316. doi: 10.1371/journal.pgph.0004316 (PMC11975090; doi:10.1371/journal.pgph.0004316)
Supplement: Attachment — Submitted filename: Author response.docx [file pgph.0004316.s006.docx]

Authors’ Responses to Reviewers Comments

Dear Academic Reviewers of PLOS Global Public Health

“Pregnancy Loss and Its Predictors among Ever Pregnant Women in Sub-Saharan Africa: Multilevel Mixed Effect Negative Binomial Regression”

Dear PLOS Global Public Health Reviewers;

We are thankful for your constructive comments. We have looked at the comments and have revised our paper accordingly. We hope our paper improved as a result of incorporating the reviewer’s and academic editor’s comments and suggestions. Here are the authors’ responses to the comments.

Please find for your kind consideration the following:

1.	Revised manuscript without highlight

2.	A revised paper with highlight

3.	A rebuttal letter that responds to each point raised by the academic reviewer.

The point-by-point responses of authors are written by hoping these changes would meet with your favorable consideration, we are happy to hear if there are more comments and suggestions. Please do not hesitate to let us know if you have any questions.

Yours Sincerely

Mr. Abel Endawkie Correspondence Author

Department of Epidemiology and Biostatistics School of Public Health College of Medicine and Health Science Wollo University Dessie Ethiopia

Tel. 251935459310	Email address: abelendawkie@gmail.com

We have tried our best to improve it accordingly:

Point-by-point response of Authors for editor's and reviewer's comment

**Editor comment**:

I thank the authors for addressing the comments. The revised manuscript addresses most of the flagged areas. However, I have noticed some inconsistencies between the text and Table 2:

Response: Dear editor, thank you so much for your kind words about our work! We truly appreciate your thoughtful feedback. We've carefully considered your recommendations and made necessary revisions to enhance our manuscript. Your insights have been invaluable, and we look forward to your thoughts on the changes and hoping these changes would meet with your favorable consideration. Thank you again for your encouraging and positive feedback.

Comment 1: In line 245, the median number of abortions is reported as 7.9 (7.6, 8.1), but Table 2 presents the mean. Similar inconsistencies are observed for miscarriage and stillbirth.

Response 1: Dear editor, thank you so much for your kind words about our work! Actually it is median since the count data is skewed by nature and the result is median. We had revised it.

Comment 2: In lines 30 and 353, it is stated that there are three pregnancy losses for every ten pregnancies among ever-pregnant women in Sub-Saharan Africa. However, Table 2 indicates a median of 2.67 pregnancy losses per woman. I could not find any data in the results section supporting the claim of 3 out of 10 pregnancies being lost.

Response 2: Dear editor, thank you so much for your kind words about our work! We had revised it.

Comment 3: I suggest revising this statement to indicate "three pregnancy losses per woman," or adding a row in Table 2 showing the percentage of pregnancies lost to support the claims in lines 30 and 353.

Response 3: Dear Editor, thank you for your valuable feedback on our manuscript. We appreciate your suggestion and have revised the statement to indicate "three pregnancy losses among ever pregnant woman." Thank you for helping us improve our work!

Comment 4: Please include the total number of pregnancies analyzed and the total number of pregnancy losses in Table 2 or in text for clarity.

Response 4: Thank you for your valuable feedback! We have made the necessary revisions. In total, we analyzed data from 235,086 who had ever been pregnant women before the survey in Sub-Saharan Africa. The highest number of pregnancy losses recorded was 9. We have ensured that this information is clearly presented in Table 2 and in the text for better clarity.

Comment 5. The column for percentages in Table 2 appears unnecessary and could be removed.

Response 5: Dear Editor, thank you for your insightful feedback regarding Table 2. We appreciate your suggestion and have removed the percentage column as recommended.

**Finally, I appreciate your kind response and genuine communication. I hope these changes will meet your favorable consideration and I am happy to hear if there are more comments and suggestions. Please do not hesitate to let me know if you have any questions.**

**I feel all of you may understand our feelings and interests.**

**I am thankful for all!!**
