# Supplementary material for: Pregnancy loss and its predictors among ever-pregnant women in Sub-Saharan Africa: Multilevel mixed effect negative binomial regression
Source: PLOS Glob Public Health. 2025 Apr 7;5(4):e0004316. doi: 10.1371/journal.pgph.0004316 (PMC11975090; doi:10.1371/journal.pgph.0004316)
Supplement: Attachment — Submitted filename: Author_response_auresp_5.docx [file pgph.0004316.s007.docx]

Authors’ Responses to Reviewers Comments

Dear Academic Reviewers of PLOS Global Public Health

“Pregnancy Loss and Its Predictors among Ever Pregnant Women in Sub-Saharan Africa: Multilevel Mixed Effect Negative Binomial Regression”

Dear PLOS Global Public Health Reviewers;

We are thankful for your constructive comments. We have looked at the comments and have revised our paper accordingly. We hope our paper improved as a result of incorporating the reviewer’s and academic editor’s comments and suggestions. Here are the authors’ responses to the comments.

Please find for your kind consideration the following:

1.	Revised manuscript without highlight

2.	A revised paper with highlight

3.	A rebuttal letter that responds to each point raised by the academic reviewer.

The point-by-point responses of authors are written by hoping these changes would meet with your favorable consideration, we are happy to hear if there are more comments and suggestions. Please do not hesitate to let us know if you have any questions.

Yours Sincerely

Mr. Abel Endawkie Correspondence Author

Department of Epidemiology and Biostatistics School of Public Health College of Medicine and Health Science Wollo University Dessie Ethiopia

Tel. 251935459310	Email address: abelendawkie@gmail.com

We have tried our best to improve it accordingly:

Point-by-point response of Authors for editor's and reviewer's comment

**Editor comment**:

Dear Dr. Endawkie, Thank you for submitting your revised manuscript to PLOS Global Public Health. I have one final comment before approving this manuscript. Since the total number of pregnancy losses is not reported in Table 2 or elsewhere, it is difficult to understand how the median number of pregnancy losses is less than the median number of abortions or miscarriages, as the latter should be subsets of pregnancy loss.

Response: Dear Editor, Thank you very much for your kind words regarding our manuscript. We sincerely appreciate your thoughtful feedback and the opportunity to clarify our findings.

To address your comment, we would like to specify that the total pooled number of pregnancy losses among ever-pregnant women in Sub-Saharan Africa (SSA) is 44,877, with a maximum of 9 pregnancy losses reported per individual. The median pregnancy loss figure we provided is calculated by considering all types of losses, including abortions, miscarriages, and stillbirths.

We understand the confusion regarding the relationship between median pregnancy losses and the median number of abortions or miscarriages. In our analysis, the median number of pregnancy losses reflects the overall count, which encompasses all three types of loss—stillbirths, abortions, and miscarriages. It is important to note that the median for abortions and miscarriages may appear higher because they are subsets of the broader category of pregnancy loss. Take into consideration that the actual median value and confidence interval for each type of pregnancy loss is not above the maximum number of pregnancy losses. This means that when we refer to the actual median value and confidence interval for each type of pregnancy loss (which includes stillbirths, miscarriages, and abortions), we emphasize that these values are derived from the data we analyzed. The median represents the middle value when all instances of a specific type of loss are arranged in order. Because the maximum number of losses reported is 9, it follows that the median and its confidence interval for each type of loss cannot exceed this maximum.

For example, the median number of miscarriages is reported as 6, this indicates that half of the individuals experienced 6 or fewer miscarriages, while the other half experienced more. However, since no one experienced more than 9 total losses, it inherently constrains the median and confidence interval for each type of loss to remain below this maximum threshold.

To clarify further, pregnancy loss in our study includes both spontaneous abortion (miscarriage) and induced abortion before 28 weeks of gestation, as well as stillbirths occurring after 28 weeks.

Thank you once again for your insightful comments and encouragement. We hope this explanation helps to clarify our findings. Thank you again for your encouraging and positive feedback.

**Finally, I appreciate your kind response and genuine communication. I hope these changes will meet your favorable consideration and I am happy to hear if there are more comments and suggestions. Please do not hesitate to let me know if you have any questions.**
